# Beliefs About Medicines and Adherence to Biologic Therapy in Patients with Moderate-to-Severe Psoriasis: A Cross-Sectional Study

**DOI:** 10.3390/medicina61112000

**Published:** 2025-11-07

**Authors:** Maja Pavić, Joško Markić, Adela Markota Čagalj, Zdenka Šitum Čeprnja, Tina Gogić Salapić, Bepa Pavlić, Petra Kuzmanić, Hannah Vasquez, Iva Bojčić, Ranka Ivanišević, Dubravka Vuković

**Affiliations:** 1 Department of Dermatovenerology, University Hospital of Split, Spinčićeva 1, 21000 Split, Croatia; pavicmajaa@gmail.com (M.P.); adela.markota@gmail.com (A.M.Č.); zsitum@kbsplit.hr (Z.Š.Č.); tgogic@kbsplit.hr (T.G.S.); bpavlic@kbsplit.hr (B.P.); iva.bojcicdu@gmail.com (I.B.); rivanisevic@kbsplit.hr (R.I.); 2 Department of Pediatrics, University Hospital of Split, Spinčićeva 1, 21000 Split, Croatia; jmarkic@mefst.hr; 3 School of Medicine, University of Split, Šoltanska 2, 21000 Split, Croatia; hannah.vasquez@mefst.hr; 4 Faculty of Humanities and Social Sciences in Split, University of Split, Poljička cesta 35, 21000 Split, Croatia; pkuzmanic@ffst.hr

**Keywords:** biologic therapy, psoriasis, adherence, BMQ-G, BMQ-S, MARS-5

## Abstract

*Background and Objectives*: Psoriasis is a chronic, immune-mediated inflammatory dermatosis affecting approximately 125 million people worldwide. Biologic therapy (BT) has significantly improved treatment outcomes for moderate-to-severe psoriasis, but real-world adherence to these therapies is not fully understood. This study aimed to evaluate adherence to BT among patients with moderate-to-severe psoriasis and examine how general and treatment-specific beliefs about BT are associated with adherence. *Materials and Methods*: This cross-sectional study was conducted from March to August 2025 at the University Hospital of Split, Croatia. A total of 122 adults with moderate-to-severe psoriasis receiving interleukin-17 or interleukin-23 inhibitors completed validated Croatian versions of the Beliefs about Medicines Questionnaire–General (BMQ-G), Beliefs about Medicines Questionnaire–Specific (BMQ-S), and the Medication Adherence Report Scale (MARS-5). Adherence was assessed using MARS-5, and beliefs were analyzed using BMQ-G, BMQ-S, and the Necessity–Concerns Framework. Correlation and group-based analyses were used to explore associations between beliefs, adherence, and sociodemographic factors. *Results*: Adherence to BT was high, with 96.7% of participants reporting that they use their medication as prescribed and 84.4% stating they always followed dosing instructions. Most participants endorsed strong necessity beliefs regarding BT; however, concerns remained common—40.2% reported worry about long-term effects and 15.6% about dependence. General beliefs about medicines influenced treatment-specific perceptions: BMQ-G Overuse scores were negatively correlated with adherence (r = −0.27; *p* = 0.003) and positively correlated with BMQ-Specific concerns. Participants classified as Distrustful (low necessity, high concerns) made up 31.1% of the cohort and had the highest Harm and Overuse scores. Lower educational attainment was associated with stronger negative beliefs about medicines. *Conclusions*: Although adherence to BT was high, a considerable proportion of patients expressed residual concerns, especially those with more negative general beliefs about medicines. These findings underscore the importance of assessing medication beliefs in routine care. Tailored education and communication strategies may help address concerns and support long-term adherence to BT in psoriasis.

## 1. Introduction

Psoriasis is a chronic, immune-mediated inflammatory skin disease characterized by the formation of well-demarcated erythematous and scaly plaques on various parts of the body, most commonly the scalp, elbows, knees, and trunk [1]. It is estimated to affect approximately 125 million people worldwide, with prevalence estimates ranging from 0.09% to 11.43% and a growing body of evidence indicating a steady rise in incidence over recent decades [2,3]. In Croatia, psoriasis affects an estimated 1.3% to 1.6% of the population—corresponding to nearly 57,000 individuals—and has a considerable impact on quality of life, work ability, and healthcare utilization [4]. Beyond its cutaneous manifestations, psoriasis is associated with a range of systemic comorbidities such as psoriatic arthritis, cardiovascular disease, metabolic syndrome, and depression, which together contribute to considerable physical, psychological, and socioeconomic burden of disease [5].

Disease severity is typically classified as mild, moderate, or severe using validated instruments such as the Psoriasis Area and Severity Index (PASI). While mild psoriasis can often be effectively managed with topical agents, moderate-to-severe forms are associated with substantial impairment in quality of life and usually necessitate systemic treatment to achieve effective and sustained disease control. Such treatments include conventional systemic agents or biologic therapy (BT) [6]. The advent of biologics has transformed the therapeutic landscape of moderate-to-severe psoriasis, particularly agents targeting interleukin-17 (IL-17) and interleukin-23 (IL-23), which have demonstrated rapid onset of action, long-term efficacy, and high rates of skin clearance [7,8].

Adherence to treatment, defined as the consistency with which patients follow prescribed treatment regimens, plays a critical role in the effective management of chronic diseases [9]. Patients who perceive their treatment as necessary and have fewer concerns about its safety are generally more likely to adhere, as shown across various chronic disease settings. In chronic dermatological conditions, adherence is influenced not only by clinical factors but also by patients’ psychological and perceptual responses, particularly their beliefs about medicines [10,11]. In psoriasis, maintaining long-term adherence is often challenging, with reported rates ranging from 31.5–62% across both topical and systemic therapies [12]. Although biologic agents have significantly improved treatment outcomes for moderate-to-severe psoriasis, real-world adherence to these therapies remains variable and not fully understood. In particular, the influence of both general and treatment-specific beliefs on adherence to BT remains insufficiently explored in this patient population. A deeper understanding of these psychological determinants is essential for optimising therapeutic outcomes and guiding clinical decision-making [13].

This study aimed to evaluate adherence to BT among patients with moderate-to-severe psoriasis treated at the University Hospital of Split and to assess how general and treatment-specific beliefs about medicines influence their adherence to BT.

## 2. Methods

### 2.1. Study Design

A cross-sectional observational study was conducted at the University Hospital of Split in Croatia, between 1 March and 31 August 2025. Participants were recruited using consecutive sampling during routine clinical visits to the Outpatient unit of the Department of Dermatovenerology. Eligible participants were adults aged 18 years or older, with a confirmed diagnosis of moderate-to-severe plaque psoriasis established by a board-certified dermatologist and currently receiving BT targeting IL-17 or IL-23. Participants were required to be capable of independently completing questionnaires and to provide written informed consent. Individuals were excluded if they had a known psychiatric or cognitive disorder that could compromise the reliability of self-reported data.

Upon enrollment, participants completed a set of questionnaires designed to assess demographic characteristics, beliefs about medicines—both general (BMQ-G) and specific (BMQ-S)—and medication adherence, measured using the Medication Adherence Report Scale (MARS-5) [14,15]. Prior to their administration, formal permission for the use of all three instruments was obtained from the original author. As the BMQ-G, BMQ-S, and MARS-5 had not previously been available in the Croatian language, they were translated and validated for use in this study. The BMQ-G was administered in its original form with minor linguistic adaptations, which were reviewed and approved by the original author. The BMQ-S was adapted to reflect BT, in agreement with the original author. For the MARS-5, a modified version was employed, in which the final item was positively phrased to reduce response bias. This adaptation was likewise approved by the original author.

Two native Croatian-speaking dermatovenereologists independently produced initial translations from English to Croatian. They subsequently reviewed their versions, discussed discrepancies, and developed a consensus translation. These versions were subsequently back-translated into English by a native English speaker who was blinded to the study’s objectives and had no access to the original versions of the questionnaires. A multidisciplinary expert panel, comprising three dermatovenereologists and one psychologist, compared the back-translations with the original questionnaires, identified discrepancies and implemented necessary adjustments to produce a finalized Croatian version of the instruments. A pilot study was subsequently conducted with a small sample of patients. All participants provided written informed consent. The time required to complete each questionnaire was recorded, and participants were invited to comment on the clarity and comprehensibility of the items. The expert panel reviewed this feedback and incorporated additional revisions as needed. Internal consistency was then assessed, yielding Cronbach’s α of 0.833 for the BMQ-G, 0.838 for the BMQ-S, and 0.769 for the MARS-5. As the objective of the study was to apply translated instruments in a clinical setting, test–retest reliability and construct validity were not assessed. The finalized, validated Croatian versions of the BMQ-G, BMQ-S, and MARS-5 were subsequently used in the main phase of the research.

Sample size calculation was based on available literature indicating a psoriasis prevalence of 1.6% among adults in Croatia and on the most recent national census data from 2021 [4,16]. Assuming a 5% margin of error and a 95% confidence level, the minimum required sample size was estimated to be 25 participants. The calculation was performed using the Epitools sample size calculator (Ausvet, available online: https://epitools.ausvet.com.au/samplesize (accessed on 10 February 2025)).

Participants were categorized into the In-favour, Mixed-feelings, Indifferent, or Distrustful groups based on their Necessity and Concern scores on the BMQ-S, in accordance with the Necessity–Concern Framework. Scores above the midpoint (≥15 for Necessity; ≥15 for Concerns) were considered high, and scores below the midpoint were considered low. Participants with high Necessity and low Concerns were categorized as “In-favour”, those with high Necessity and high Concerns as “Mixed-feelings”, those with low Necessity and low Concerns as “Indifferent”, and those with low Necessity and high Concerns as “Distrustful” [17,18].

### 2.2. Statistical Analysis

Continuous variables (age, treatment duration, BMQ-General and BMQ-Specific scores, and MARS-5 score) are summarized as medians with interquartile ranges (IQRs), and categorical variables (sex, educational attainment, employment status, marital status, healthcare profession status, type of systemic conventional therapy, type of biologic therapy, and biologic therapy switch) are presented as frequencies and percentages. Normality of continuous variables was assessed using the Shapiro–Wilk test. Group comparisons between continuous outcomes and binary categorical variables were conducted using *t* tests or point-biserial correlations, as appropriate. For comparisons involving categorical variables with more than two levels and non-normally distributed continuous outcomes, the Kruskal–Wallis test was applied, followed by Dunn’s post hoc test with Holm correction. Spearman’s rank correlation was used to assess associations between ordinal or non-normally distributed continuous variables. The relationship between treatment duration and adherence was further examined using Spearman and Pearson correlations, as well as linear regression analysis with 95% confidence intervals (CIs). Statistical significance was set at *p* < 0.05. All analyses were performed in RStudio, version 4.3.1 (RStudio, PBC, Boston, MA, USA).

## 3. Results

### 3.1. Baseline Characteristics of Participants

A total of 122 participants were included in the study, with a median age of 47.5 years (IQR = 39.0–57.0). The majority were male (64.8%) and married (68.0%) (Table 1). Secondary education was the most commonly reported level of education (58.2%). Most participants were employed at the time of the study (71.3%), and only a small proportion were healthcare professionals (5.7%). Prior to initiating BT, the most frequently used conventional treatments were methotrexate (39.2%) and phototherapy (36.3%). Anti–IL-23 agents were prescribed in 83.6% of cases, while anti–IL-17 therapy was used in 16.4%. The median duration of BT was 24.0 months (IQR = 18.0–38.0). Most participants had not switched biologic agents (86.9%), while 13.1% had changed therapy at least once.

### 3.2. Results of the BMQ-G Questionnaire

Most participants did not consider medicines to be harmful, with 84.4% disagreeing or strongly disagreeing with the statement that all medicines are poisons and 76.2% disagreeing or strongly disagreeing with the statement that medicines do more harm than good (Table 2). Regarding the statement that most medicines are addictive, 56.6% of participants disagreed or strongly disagreed and 31.1% were uncertain. For the statement that patients should occasionally interrupt treatment, 43.4% disagreed or strongly disagreed, 20.5% agreed or strongly agreed, and 36.1% were uncertain. Regarding natural remedies, 50.0% disagreed or strongly disagreed that natural remedies are safer than medicines, 19.7% agreed or strongly agreed, and 30.3% were uncertain.

Findings from the BMQ-G also showed that 45.1% disagreed or strongly disagreed with the statement that physicians prescribe too many medicines, 20.5% agreed or strongly agreed, and 34.4% were uncertain. More than half (54.1%) disagreed or strongly disagreed that physicians rely too much on medicines, while 16.4% agreed or strongly agreed and 29.5% were uncertain. For the statement that physicians would prescribe fewer medicines if they had more time with patients, 50.0% disagreed or strongly disagreed, 23.0% agreed or strongly agreed, and 27.0% were uncertain. In total, 90.2% of participants had higher scores on the Harm scale than on the Overuse scale, while 9.8 had Harm scores equal to or lower than Overuse scores.

Regarding demographic characteristics, educational attainment was negatively correlated with BMQ-G Harm scores (*p* = 0.045). No significant differences in BMQ-G scores were observed according to age, sex, marital status, employment status, or healthcare profession (*p* ≥ 0.05).

### 3.3. Results of the BMQ-S Questionnaire

A total of 93.4% of participants agreed or strongly agreed that their current health depends on BT (Table 3). For the statement that life would be impossible without BT, 68.0% agreed or strongly agreed, 14.8% were uncertain, and 17.2% disagreed or strongly disagreed. In addition, 74.5% agreed or strongly agreed that without the medicine they would be very ill, and 77.9% agreed or strongly agreed that their future health will depend on it. For the statement that BT protects them from worsening of disease, 89.4% agreed or strongly agreed, while 10.6% expressed uncertainty or disagreement.

While a total of 20.5% of participants agreed or strongly agreed that they worry about having to take the medicine, 23.8% were uncertain, and 55.7% disagreed or strongly disagreed. Concerns about long-term effects were reported by 40.2% of participants, while 36.9% disagreed or strongly disagreed and 23.0% were uncertain. A minority (14.7%) agreed or strongly agreed that the medicine is a mystery to them, whereas 64.7% disagreed or strongly disagreed. Only 2.4% reported that the medicine disrupts their life, compared with 91.8% who disagreed or strongly disagreed. Dependence was a concern for 15.6% of participants, while 70.5% disagreed or strongly disagreed. No participants agreed or strongly agreed that the medicine causes unpleasant side effects; instead, 85.3% disagreed or strongly disagreed, and 14.8% were uncertain.

The Necessity–Concerns framework indicated a strong perceived need for BT. A total of 91.0% of participants scored above the midpoint on the Necessity scale (>15), whereas only 12.3% scored above the midpoint on the Concerns scale (>18) (Table 4). The Necessity–Concerns differential was positive in 82.8% of participants, indicating that perceived necessity exceeded concerns, while it was neutral in 2.5% and negative in 14.8%.

Regarding demographic characteristics, divorced participants reported higher BMQ-S Concerns scores compared with married participants (*p* = 0.046). No significant differences in BMQ-G scores were observed according to age, sex, educational attainment, employment status, or healthcare profession (*p* ≥ 0.05).

### 3.4. Results of the MARS-5

Most participants reported rarely or never forgetting to take their medication (85.2%) and not altering the prescribed dosage (98.3%). The majority also indicated that they did not skip doses when feeling better (91.8%). Overall, 96.7% of participants reported taking their medication as prescribed, with 84.4% stating they always followed dosing instructions. A total of 16.4% of participants reported taking the medication only when needed, and an additional 12.3% reported doing so occasionally (Table 5).

The median MARS-5 score was 20.0 (IQR = 17.0–21.0), with 37% of participants achieving the maximum score of 25. No significant correlation was observed between treatment duration and MARS-5 scores (Spearman ρ = −0.024; *p* = 0.796). Regarding demographic characteristics, older participants had significantly higher MARS-5 scores (*p* = 0.022). Retired participants also demonstrated higher adherence compared with those who were employed (*p* = 0.018). No significant differences in MARS-5 scores were observed by sex, marital status, educational attainment, healthcare profession or type of BT (*p* ≥ 0.05).

### 3.5. Correlation Between BMQ-G, BMQ-S, and MARS-5

Pearson correlations among scales showed that BMQ-G Overuse was positively correlated with BMQ-G Harm (r = 0.58; *p* < 0.001) and with BMQ-S Concerns (r = 0.34; *p* < 0.001) (Table 6). BMQ-G Harm was positively correlated with BMQ-S Concerns (r = 0.46; *p* < 0.001) and negatively correlated with BMQ-S Necessity (r = −0.18; *p* = 0.043). BMQ-S Necessity was negatively correlated with BMQ-S Concerns (r = −0.39; *p* < 0.001). MARS-5 scores were negatively correlated with BMQ-G Overuse (r = −0.27; *p* = 0.003).

### 3.6. Necessity–Concern Framework Group Classification and BMQ Subscale Scores

According to the Necessity–Concern framework, 40 participants (32.8%) were classified as In-favour, 23 (18.9%) as Mixed-feelings, 21 (17.2%) as Indifferent, and 38 (31.1%) as Distrustful. Median Harm scores were highest in the Distrustful group (Md = 10.0, IQR = 9.0–11.8) and lowest in the In-favour group (Md = 7.5, IQR = 5.8–9.0). Median Overuse scores were likewise highest in the Distrustful group (Md = 11.0, IQR = 9.3–13.0) and lowest in the In-favour group (Md = 9.0, IQR = 7.0–11.0) (Table 7).

## 4. Discussion

In this cross-sectional study of patients with moderate-to-severe psoriasis receiving BT, most participants reported high adherence, with over one-third achieving the maximum score on the MARS-5. These findings are consistent with previous studies reporting high adherence to BT [12], likely due to the favorable efficacy and tolerability of biologics and structured follow-up in a tertiary care setting. In contrast, adherence to conventional systemic therapies has been reported to be substantially lower, often ranging from 23–50% [19]. Nevertheless, proportion of 16.4% of participants reported taking medication only when they perceived it necessary, and an additional 12.3% did so occasionally, which could compromise long-term disease control.

Our findings indicate that older and retired participants reported higher MARS-5 scores, reflecting greater adherence to BT. These findings align with evidence from other populations with chronic illnesses, where older age and retirement are associated with more consistent medication use, likely reflecting more stable daily routines and fewer competing responsibilities [20]. We also examined whether adherence varied by type of BT. Adherence rates did not significantly differ between patients treated with IL-17 or IL-23 inhibitors. However, due to the predominance of IL-23 users in our sample, the ability to detect differences by biologic class was limited, and further studies with more balanced groups are needed to explore this relationship. No associations were observed between adherence and sex, educational attainment, or healthcare profession status. However, divorced participants reported significantly higher BMQ-S Concern scores, indicating that personal and social circumstances may shape treatment beliefs.

Analysis of the BMQ-General revealed belief patterns broadly consistent with the high adherence observed. Most participants did not endorse the view that medicines are inherently harmful, as reflected by strong disagreement with statements such as “all medicines are poisons” and “medicines do more harm than good.” Compared with reports from other chronic illness populations—where such beliefs are more common and associated with lower adherence [21,22]—these findings suggest that general harm beliefs were less prominent in our sample and may have supported stronger adherence to BT. However, some uncertainty persisted, particularly regarding the potential for addiction, the acceptability of interrupting treatment, and the perceived safety of natural remedies. These beliefs, which remain present even among adherent patients, mirror prior findings showing that 20–40% of individuals with chronic conditions hold similar views [23,24]. In addition, while nearly half of the participants rejected the idea that physicians overprescribe medications, approximately 20% agreed, and one-third were uncertain. Similar patterns have been reported in patients with ankylosing spondylitis, hypertension and type 2 diabetes, where stronger beliefs about medication overuse and harm have been associated with lower adherence [25,26,27]. These findings underscore the importance of addressing lingering concerns about medication safety, necessity, and overuse through targeted education and shared decision-making to support long-term adherence. In addition, our finding that higher educational attainment was associated with lower perceptions of medicines as harmful in the BMQ-General further supports the connection between education level and more favorable treatment beliefs. This is consistent with prior studies in type 2 diabetes or studies examining health literacy, which have shown that lower educational attainment and limited health literacy are associated with stronger harm beliefs and reduced adherence [28,29]. Together, these findings highlight the role of education in shaping general beliefs about medicines and underscore the importance of tailored educational strategies to address negative perceptions and support adherence.

The BMQ-Specific findings suggest that participants held strong beliefs in the necessity of BT, viewing it as essential for maintaining health, preventing disease progression, and safeguarding future well-being. The predominance of a positive Necessity–Concerns differential further indicates that, for most patients, the perceived benefits of BT outweighed any reservations. These findings are consistent with those of Otero et al., who reported that most patients with psoriasis receiving biologics endorsed high necessity and low concern beliefs, in contrast to patients treated with methotrexate, who more frequently expressed ambivalence [30]. Despite this favorable necessity profile, residual concerns persisted. A substantial proportion of participants expressed worries about the long-term effects of BT, and a smaller but notable group reported concerns about becoming dependent on their medication. Although slightly higher than in earlier studies, these concern levels are consistent with trends in comparable populations. For instance, Hannech et al. found that 24% of patients with immune-mediated inflammatory diseases treated with biologic DMARDs were concerned about long-term effects, and 16% reported concerns about dependence [31]. These findings highlight that even among patients who strongly endorse the necessity of BT, concerns about safety and long-term use persist, underscoring the importance of ongoing communication, monitoring, and education to support confidence in treatment and sustain adherence over time.

Our analysis of the interrelationships between the BMQ-G, the BMQ-S, and the MARS-5 revealed several important patterns. Within the BMQ-G, Overuse scores were strongly and positively correlated with Harm scores, indicating that perceptions of overprescription often co-occurred with beliefs that medicines are inherently harmful. These general attitudes extended to treatment-specific perceptions: both Overuse and Harm scores were positively correlated with BMQ-S Concerns, suggesting that skepticism about medicines in general translated into greater worries about BT. In contrast, Harm scores were negatively correlated with Necessity, meaning that participants with stronger harm beliefs were less likely to perceive BT as essential. When examining adherence, only Overuse beliefs showed a significant negative correlation with MARS-5 scores, underscoring the impact of perceived prescribing excess on treatment behavior. Together, these findings underscore the interconnectedness of general and treatment-specific beliefs and suggest that unfavorable general attitudes may indirectly undermine adherence by lowering perceived necessity and amplifying concerns. These associations emerged despite generally high adherence, indicating that even subtle negative beliefs can influence treatment behavior. Similar patterns have been documented in other chronic conditions, including diabetes and ankylosing spondylitis, where stronger Overuse and Harm beliefs are associated with reduced adherence [23,25]. In our study, the conditional non-adherence reported by 16.4% of participants—taking medication only when they perceived it necessary—may reflect the influence of such skeptical beliefs. This finding underscores the importance of identifying and addressing these attitudes in clinical practice to support sustained adherence.

Classification of participants according to the Necessity–Concerns framework revealed meaningful differences that may help explain variations in treatment engagement. In our cohort, 31.1% of participants were classified as Distrustful, characterized by low perceived necessity and high concerns, while 32.8% were categorized as In-favour, reflecting high necessity and low concerns. Median BMQ-General Harm and Overuse scores were highest among the Distrustful group and lowest among those In-favour, suggesting that general skepticism about medicines is closely aligned with negative attitudes toward BT. Despite high overall adherence, nearly one-third of participants fell into the Distrustful category, a belief profile commonly associated with nonadherence, as shown in previous studies [17,21,32]. Our findings extend this work by showing that individuals in the Distrustful group not only harbor concerns about their specific treatment but also hold broader negative beliefs about medicines, including perceptions of overuse and inherent harm, reflecting a more pervasive mistrust of pharmacotherapy. These findings have important implications for clinical care. Identifying patients with Distrustful belief patterns may enable tailored, trust-building interventions that address both general medication skepticism and treatment-specific concerns. In practice, such patients can be recognized through brief, structured questions exploring perceived necessity and concerns about medicines. Early identification allows clinicians to tailor communication, reinforce the rationale for treatment, and build trust through shared decision-making. This is particularly relevant in the context of BT, where adherence is essential for sustained disease control and where patients may be balancing perceived treatment risks against uncertain long-term outcomes.

Our study has several limitations. Firstly, its cross-sectional design precludes conclusions about causality between beliefs and adherence. Secondly, adherence and beliefs about medicines were assessed using validated self-report instruments. While such measures may be subject to certain biases, they are widely used and recognized as reliable in adherence and belief-related research. Thirdly, the modest sample size may have limited power to detect some associations. Finally, factors beyond the individual level, such as access to care, healthcare system organization, or environmental barriers, were not addressed in this study, but may also influence adherence to biologic therapy and should be addressed in future research.

## 5. Conclusions

Among patients with moderate-to-severe psoriasis receiving biologic therapy, adherence was generally high, and most participants expressed strong beliefs in the necessity of treatment. However, a meaningful proportion also reported concerns related to long-term safety and dependence, particularly those with more negative general beliefs about medicines. These findings suggest that even in the context of effective therapies, such as BT, underlying skepticism may persist and influence treatment behavior. Furthermore, the association between lower educational attainment and stronger beliefs about medication harm and overuse highlights the role of health literacy in shaping perceptions of BT. Addressing these beliefs through tailored education and clear communication may be essential to maintaining long-term adherence, especially for patients with limited formal education or greater mistrust of pharmacologic treatments. The presence of conditional adherence and Distrustful belief profiles in a considerable portion of the sample underscores the need for proactive, belief-informed care. Routine assessment of medication beliefs may help identify patients at risk of nonadherence, enabling earlier and more effective interventions to prevent treatment failure. In the long term, such strategies may help reduce disease progression, minimize the need for switching or intensifying therapy, and support the cost-effective use of BT in routine care.

## Figures and Tables

**Table 1:** Baseline sociodemographic and clinical characteristics of participants (*N* = 122).

Baseline Characteristics	*N* (%)
**Marital Status**	
Married	83 (68.0)
Divorced	7 (5.7)
Widowed	2 (1.6)
Cohabiting	6 (4.9)
Never married	24 (19.8)
**Educational Attainment**	
Primary school	6 (4.9)
Secondary school	71 (58.2)
Post-secondary non-tertiary education	23 (18.9)
University degree	19 (15.6)
Master’s/Doctorate	3 (2.4)
**Employment Status**	
Employed	87 (71.3)
Unemployed	13 (10.7)
Retired	22 (18.0)
**Healthcare Professional**	
Yes	7 (5.7)
No	115 (94.3)
**Previous systemic conventional therapy**	
Phototherapy	86 (36.3)
Methotrexate	93 (39.2)
Acitretin	45 (19.0)
Cyclosporine	13 (5.5)
**Current Biologic Therapy**	
Anti-IL-23	102 (83.6)
Anti-IL-17	20 (16.4)
**Biologic therapy switch**	
Yes	16 (13.1)
No	106 (86.9)

**Table 2:** Responses to the Beliefs about Medicines Questionnaire–General (BMQ-G).

Item	Strongly Agree *N* (%)	Agree *N* (%)	Uncertain *N* (%)	Disagree *N* (%)	Strongly Disagree *N* (%)
Physicians use too many medications.	2 (1.6)	23 (18.9)	42 (34.4)	39 (32.0)	16 (13.1)
Physicians rely too much on medications.	2 (1.6)	18 (14.8)	36 (29.5)	39 (32.0)	27 (22.1)
If physicians had more time with patients, they would prescribe fewer medications.	8 (6.6)	20 (16.4)	33 (27.0)	39 (32.0)	22 (18.0)
Patients who take medications should occasionally stop treatment for a while.	2 (1.6)	23 (18.9)	44 (36.1)	33 (27.0)	20 (16.4)
Most medications are addictive.	4 (3.3)	11 (9.0)	38 (31.1)	45 (36.9)	24 (19.7)
All medications are poisons.	0 (0.0)	4 (3.3)	15 (12.3)	55 (45.1)	48 (39.3)
Medications do more harm than good.	1 (0.8)	5 (4.1)	23 (18.9)	56 (45.9)	37 (30.3)
Natural remedies are safer than medications.	4 (3.3)	20 (16.4)	37 (30.3)	37 (30.3)	24 (19.7)

**Table 3:** Responses to the Beliefs about Medicines Questionnaire–Specific (BMQ-S).

Item	Strongly Agree *N* (%)	Agree *N* (%)	Uncertain *N* (%)	Disagree *N* (%)	Strongly Disagree *N* (%)
My health currently depends on biologic therapy.	67 (54.9)	47 (38.5)	3 (2.5)	2 (1.6)	3 (2.5)
My life would be impossible without biologic therapy.	40 (32.8)	43 (35.1)	18 (14.8)	18 (14.8)	3 (2.5)
Without biologic therapy I would be very ill.	48 (39.3)	43 (35.2)	20 (16.4)	8 (6.6)	3 (2.5)
My future health will depend on biologic therapy.	45 (36.9)	50 (41.0)	21 (17.2)	5 (4.1)	1 (0.8)
Biologic therapy protects me from worsening of my disease.	79 (64.8)	30 (24.6)	5 (4.1)	2 (1.6)	6 (4.9)
It concerns me that I must take biologic therapy.	4 (3.3)	21 (17.2)	29 (23.8)	37 (30.3)	31 (25.4)
Sometimes I worry about the long-term effects of biologic therapy.	10 (8.2)	39 (32.0)	28 (23.0)	23 (18.9)	22 (17.9)
Biologic therapy is unclear to me.	2 (1.6)	16 (13.1)	25 (20.5)	41 (33.6)	38 (31.2)
Biologic therapy disrupts my life.	2 (1.6)	1 (0.8)	7 (5.7)	48 (39.3)	64 (52.6)
Sometimes I worry that I will become too dependent on biologic therapy.	5 (4.1)	14 (11.5)	17 (13.9)	40 (32.8)	46 (37.7)
Biologic therapy gives me unpleasant side effects.	0 (0.0)	0 (0.0)	18 (14.8)	44 (36.1)	60 (49.1)

**Table 4:** Necessity and concerns scores from the Beliefs about Medicines Questionnaire–Specific (BMQ-S).

Category	*N* (%)
**Necessity**	
≤15	11 (9.0)
>15	111 (91.0)
**Concerns**	
≤18	107 (87.7)
>18	15 (12.3)
**Necessity–Concerns**	
<0 (negative)	18 (14.8)
=0 (neutral)	3 (2.5)
>0 (positive)	101 (82.8)

**Table 5:** Results of the Medication Adherence Report Scale (MARS-5).

Item	Always *N* (%)	Often *N* (%)	Sometimes *N* (%)	Rarely *N* (%)	Never *N* (%)
I forget to take my medication.	0 (0.0)	0 (0.0)	18 (14.8)	31 (25.4)	73 (59.8)
I alter the dose of my medication.	0 (0.0)	0 (0.0)	2 (1.6)	17 (13.9)	103 (84.5)
I take my medication only when I feel like I need them.	17 (13.9)	3 (2.5)	15 (12.3)	17 (13.9)	70 (57.4)
I miss out on a dose if I feel better.	0 (0.0)	2 (1.6)	8 (6.6)	24 (19.7)	88 (72.1)
I use my medication as prescribed.	103 (84.5)	15 (12.3)	2 (1.6)	0 (0.0)	2 (1.6)

**Table 6:** Correlations Between the Beliefs About Medicines Questionnaires—General and Specific (BMQ-G and BMQ-S) and the Medication Adherence Report Scale (MARS-5).

Correlation	r *	*p* Value
MARS-5 vs. BMQ-G Overuse	−0.269	0.003
MARS-5 vs. BMQ-G Harm	−0.082	0.370
MARS-5 vs. BMQ-S Concerns	0.011	0.905
MARS-5 vs. BMQ-S Necessity	0.085	0.352
BMQ-G Overuse vs. BMQ-G Harm	0.583	<0.001
BMQ-G Overuse vs. BMQ-S Concerns	0.337	<0.001
BMQ-G Overuse vs. BMQ-S Necessity	−0.172	0.058
BMQ-G Harm vs. BMQ-S Concerns	0.462	<0.001
BMQ-G Harm vs. BMQ-S Necessity	−0.184	0.043
BMQ-S Necessity vs. BMQ-S Concerns	−0.394	<0.001

* Pearson coefficient.

**Table 7:** Beliefs About Medicines Questionnaires subscale scores by Necessity–Concern framework group.

BMQ Subscale	In-Favour (*N* = 40) Md (IQR)	Mixed-Feelings (*N* = 23) Md (IQR)	Indifferent (*N* = 21) Md (IQR)	Distrustful (*N* = 38) Md (IQR)
Necessity	24.5 (22.0–25.0)	23.0 (22.0–25.0)	19.0 (17.0–21.0)	18.0 (16.0–20.0)
Concern	8.5 (6.0–11.0)	16.0 (13.50–17.50)	11.0 (8.0–12.00)	17.0 (15.0–19.0)
Necessity–Concern Differential	15.5 (13.0–19.0)	7.0 (5.00–10.0)	9.0 (6.0–10.0)	1.0 (−2.0–3.0)
Harm	7.5 (5.8–9.0)	10.0 (8.0–12.0)	8.0 (7.0–10.0)	10.0 (9.0–11.8)
Overuse	9.0 (7.0–11.0)	11.0 (9.0–13.0)	11.0 (8.0–12.0)	11.0 (9.3–13.0)

Abbreviations: Md—median, IQR—interquartile range.

## Data Availability

The authors have complete control of all primary data and agree to allow the journal to review the data on request.
